# Comparison of the Diagnostic Performance of Novel Slim Biopsy Forceps with Conventional Biopsy Forceps for Biliary Stricture: A Multicenter Retrospective Study

**DOI:** 10.3390/jpm11010055

**Published:** 2021-01-17

**Authors:** Eun Suk Jung, Se Woo Park, Jung Hee Kim, Jang Han Jung, Min Jae Yang, Da Hae Park

**Affiliations:** 1 Division of Gastroenterology, Department of Internal Medicine, Hallym University Dongtan Sacred Heart Hospital, Hallym University College of Medicine, 7, Keunjaebong-gil, Hwaseong-si, Gyeonggi-do 18450, Korea; esjung@hallym.or.kr (E.S.J.); jungheekim@hallym.or.kr (J.H.K.); con2000@hallym.or.kr (J.H.J.); dahaepark82@gmail.com (D.H.P.); 2 Department of Gastroenterology, Ajou University School of Medicine, 164, Worldcup-ro, Yeongtong-gu, Suwon-si, Gyeonggi-do 16499, Korea; creator1999@hanmail.net

**Keywords:** endoscopic retrograde cholangiopancreatography, biliary, stricture, forceps, diagnosis

## Abstract

Novel slim biopsy forceps provide some technical advantages to facilitate a more accurate diagnosis, although we are not aware of any comparative studies. Therefore, we compared tissue acquisition and diagnostic accuracy between novel slim biopsy forceps and conventional biopsy forceps in cases with a biliary stricture. We reviewed 341 patients who underwent endoscopic retrograde cholangiopancreatography for the histological confirmation of biliary stricture at two tertiary hospitals between 2013 and 2020. The primary endpoint was the forceps’ diagnostic accuracies. We included 276 patients who underwent biopsy using the novel forceps (*n* = 130) or conventional forceps (*n* = 146). The novel forceps provided 81.7% sensitivity, 100.0% specificity, positive-predictive value (PPV) of 100.0%, and negative-predictive value (NPV) of 57.8%, with an accuracy of 85.4% when the diagnosis by endobiliary biopsy included suspected or positive malignancy. The conventional forceps provided 61.7% sensitivity, 100.0% specificity, PPV of 100.0%, and NPV of 36.1%, with an accuracy of 68.5%. Only novel forceps use was significantly associated with an accurate diagnosis (odds ratio: 2.70, 95% confidence interval: 1.52–5.00). There were no significant inter-group differences in the procedure-related rates of adverse events. Endobiliary biopsy using novel forceps offered better diagnostic performance and more acceptable procedure-related adverse events than conventional forceps.

## 1. Introduction

The initial strategy for diagnosing biliary stricture involves minimally invasive imaging modalities, such as multi-detector-computed tomography (MDCT) and magnetic resonance imaging (MRI), with or without magnetic resonance cholangiopancreatography (MRCP) and endoscopic ultrasonography (EUS) [1]. Nevertheless, an accurate diagnosis for biliary strictures should only be established by histologic evaluation, which is essential for making a therapeutic decision, including surgical resection or adjuvant therapy [2]. Biliary strictures are also challenging to accurately diagnose via endoscopic retrograde cholangiopancreatography (ERCP), as physicians tend to weigh the malignant potential over benign etiologies, and the results are often influenced by the anatomical level, the angulation of the bile duct, and the degree of stricture [3].

In addition to aspiration cytology or brush cytology, which may not theoretically provide a sufficient specimen, endobiliary forceps biopsy can be used to collect a histological core specimen that extends deep into the epithelium [4]. However, a select series that evaluated endobiliary forceps biopsies for malignant biliary strictures revealed sensitivity values of 43–82% and negative predictive values of 31–82% [5,6,7,8,9,10,11,12]. A recent meta-analysis [13] revealed that the pooled sensitivity and specificity of an endobiliary forceps biopsy for the diagnosis of malignant biliary stricture were 48.1% and 99.2%, respectively. Although a combination of endobiliary forceps biopsy and brush cytology modestly increased the pooled sensitivity and specificity (59.4% and 100%, respectively), the diagnosis of biliary stricture is still challenging. These disappointing results may be related to several critical limitations of conventional biopsy forceps. First, the relatively small forceps jaw may not collect a sufficient amount of specimen, which can necessitate subsequent reinterventions, including ERCP with endobiliary biopsy [11]. Second, the duct below the stricture is often angulated because of the tumor’s presence, making it difficult to advance the forceps to the target lesion. Third, sclerosing-type tumors can induce a “bounce off” effect, whereby the forceps slip suddenly on the tumor’s hard surface and do not collect a sufficient amount of specimen [5]. Yamamoto et al. [14] recently reported that novel slim biopsy forceps, which have a thin/soft shaft and radial jaws, permit advancement along the bile duct wall and reliably collect specimens for diagnosing biliary strictures [14]. Nevertheless, it is unclear whether the theoretical benefits of the novel slim biopsy forceps provide superior real-world outcomes relative to conventional biopsy forceps, and we are unaware of any comparative studies. Therefore, we retrospectively compared the tissue acquisition and diagnostic abilities of the novel slim biopsy forceps and conventional biopsy forceps in cases with biliary strictures.

## 2. Materials and Methods

### 2.1. Study Population

We retrospectively reviewed data from patients who underwent therapeutic ERCP for biliary stricture at Hallym University Dongtan Sacred Heart Hospital and Ajou University Hospital in South Korea between 2013 and 2020. Among them, patients with any documentation of histologic confirmation under ERCP but no other recognized interventions such as stone removal were recruited, and only patients who were followed up for more than 6 months post-ERCP were included. Exclusion criteria were (1) patients with strictures that were located deep in the intrahepatic duct above the main ductal branch, which made it impossible to deliver forceps; (2) patients who underwent other primary diagnostic modalities such as percutaneous transhepatic cholangioscopy (PTCS) or direct peroral cholangioscopy (DOC); (3) patients who underwent other rescue modalities such as PTCS or DOC; and (4) patients with incomplete medical records. Patients transferred from another institution after histologic confirmation for biliary stricture were also excluded due to inadequate documentation. In study institutions, endoscopists used conventional forceps for histologic confirmation under ERCP until February 2016, while we used novel slim biopsy forceps for all cases after March 2016 because they had been introduced and were clinically available on the market from that point. The level of biliary stricture was classified according to the confluence of the hepatic ducts as extrahepatic and perihilar stricture, based on ERCP findings at the time of the primary diagnosis. We also attempted to clarify the strictures’ etiologies using information from electronic medical records for cases within >6 months of follow-up. The requirement for informed consent was waived based on the retrospective design, and the retrospective protocol was approved by the appropriate institutional review boards (approval number: HDT 2020-04-007).

### 2.2. Endpoints and Outcomes

The primary endpoint was the diagnostic accuracies of procedures using the novel or conventional forceps. The secondary endpoints were variables that affected the diagnostic accuracy in a logistic regression model, diagnostic performance parameters (including sensitivity and specificity), and procedure-related adverse events (AEs).

A final confirmed diagnosis of benign or malignant disease was based on the following: (a) a definite pathological diagnosis based on the surgical specimen from patients who underwent surgical resection, (b) disease-specific death, or (c) no signs of disease progression or regression during a ≥6-month follow-up period with clinical observation and imaging evaluation of suspected benign strictures [15]. Lesions were initially categorized as positive malignancy, suspected malignancy, or negative based on the histological findings from the endobiliary forceps biopsy. Pathologically negative cases were classified as normal bile duct epithelium, inflammatory atypia, reactive change, or non-diagnostic results, such as inadequate material or insufficient material [16]. According to the categorization of final confirmed diagnosis and initial pathology results, patients were classified into 4 groups: (1) a true-positive diagnosis was defined as a final diagnosis of malignancy in cases that were initially classified as positive or suspected malignancy; (2) a false-positive diagnosis was defined as a final diagnosis of benign disease after clinical follow-up in cases with positive or suspected malignancy; (3) a true-negative diagnosis was defined as a final diagnosis of benign disease in cases that were initially classified as negative results; (4) a false-negative diagnosis was defined as a final diagnosis of malignant disease in cases that were initially classified as negative results.

### 2.3. Endoscopic Procedures for ERCP

Cannulation of the extrahepatic duct (EHD) was attempted using a standard catheter and/or a pull-type sphincterotome (Clever-cut^®^; Olympus, Tokyo, Japan) under guidewire assistance (wire-assisted cannulation). The contrast medium was only injected when the endoscopist confirmed that selective deep cannulation had been achieved using a guidewire. A rescue infundibulotomy was attempted if the cannulation was difficult. A primary infundibulotomy with a needle knife was attempted, without any attempted cannulation, if the patient had a prominent ampulla of Vater that was expected to be difficult to cannulate [17]. When a guidewire was inadvertently inserted into the pancreatic duct and failed to cannulate the EHD, the double guidewire method was occasionally used. In these cases, the second guidewire was reloaded and reinserted through the working channel of the scope to cannulate the EHD, while the first guidewire remained in the pancreatic duct [18].

**Figure 1:**
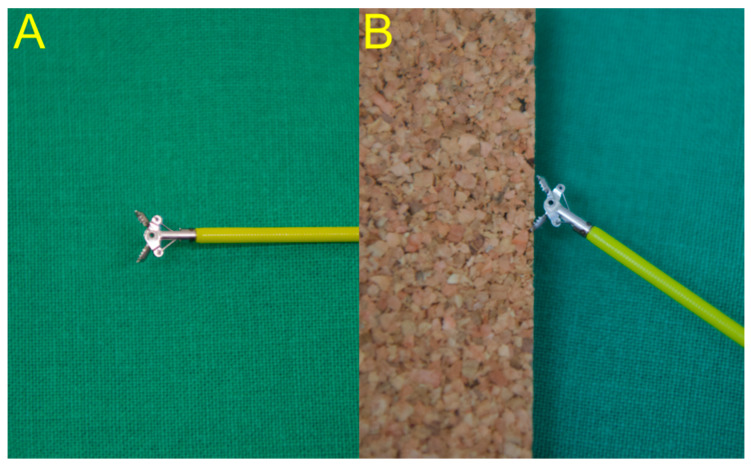
(**A**) The novel forceps have jaws that can open to a relatively wide angle. (**B**) The swing function can permit tangential biopsy even in an oblique approach angle, which minimizes the “bounce off” effect.

**Figure 2:**
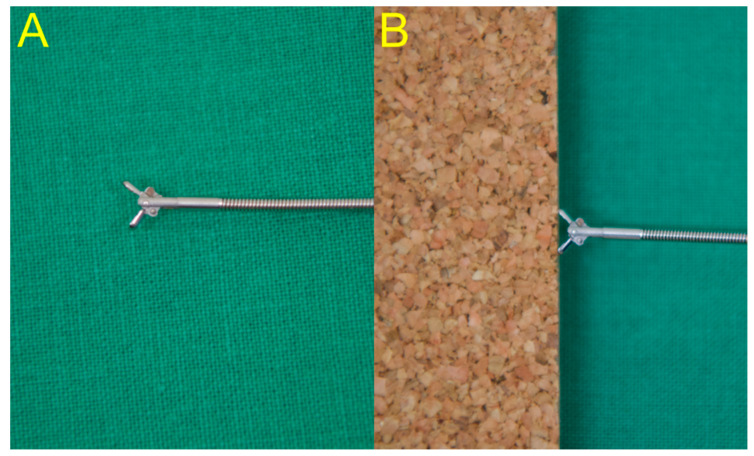
(**A**) The conventional forceps have a relatively small forceps jaw, which cannot collect a sufficient amount of specimen. (**B**) The conventional forceps do not have the swing function; thus, they can only permit the vertical approach.

After selective deep cannulation of the EHD, the location and length of the biliary stricture were confirmed under fluoroscopic guidance using contrast medium. Endoscopic sphincterotomy (EST) was then performed over the guidewire using the pull-type sphincterotome. If the clinical presentation of the patients was unstable due to sepsis derived from acute cholangitis with biliary obstruction, endoscopists firstly performed EST with only biliary stent placement for emergency biliary decompression. Then, they sequentially performed endobiliary biopsy in the next session for histologic confirmation after stabilization of the patient’s condition. The biopsy forceps were then advanced through the opened major papilla to sit as close as possible to the distal end of the stricture, the jaws were opened, and the opened forceps were advanced gently to sit against the distal end of the biliary stricture (Figure 1: novel forceps; Figure 2: conventional forceps). The specimen was obtained by closing the forceps in this position, and then it was fixed in 10% formalin [19]. The biliary stent type and length were selected based on the endoscopist’s judgment according to the level, degree, and length of the biliary stricture.

**Figure 3:**
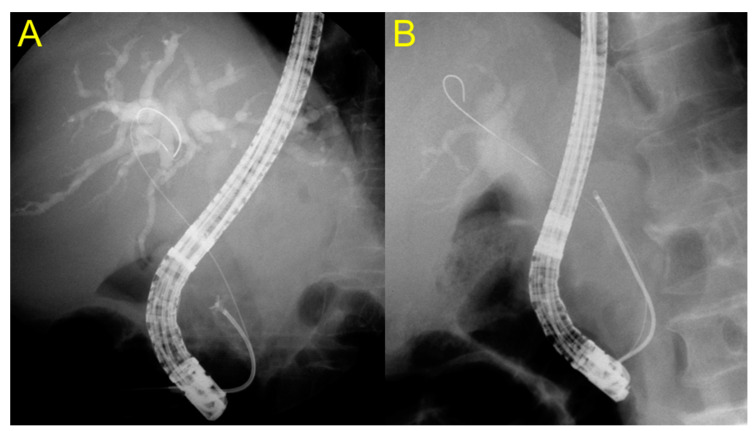
(**A**) The opened novel forceps were advanced gently to sit against the distal end of the biliary stricture. (**B**) The specimen was obtained by closing the conventional forceps in the distal end of the biliary stricture.

Endobiliary biopsy was performed using novel slim biopsy forceps that were developed for pediatric patients (Figure 3A; Radial Jaw 4P, Boston Scientific, Boston, MA, United States) or using conventional slim biopsy forceps (Figure 3B; Olympus FB-19K). The novel slim biopsy forceps have radial jaws with a swing function to permit effective tissue acquisition via an oblique approach angle.

### 2.4. Statistical Analyses

Continuous variables were presented as mean and standard deviation and were compared using Student’s *t*-test. Categorical variables were presented as a number (percentage) and were compared using the χ^2^ test. Factors that were associated with diagnostic accuracy were identified via logistic regression analysis, and variables with univariable *p*-values of <0.2 were incorporated into the multivariable analysis [20,21]. The diagnostic sensitivity, specificity, accuracy, positive predictive value (PPV), and negative predictive value (NPV) were evaluated for both forceps. The diagnostic performance was calculated by the definition of malignancy for endobiliary biopsy: (1) positive malignancy and suspected malignancy, and (2) only positive malignancy. All tests were two-tailed, and *p*-values of <0.05 were considered statistically significant. All analyses were conducted using R software (version 4.0.2; R Foundation for Statistical Computing, Vienna, Austria).

## 3. Results

### 3.1. Study Population and Baseline Characteristics

During the study period, 341 patients underwent therapeutic ERCP for histological confirmation of biliary stricture. Patients with strictures that were located deep in the intrahepatic duct above the main ductal branch, which made it impossible to deliver forceps (n = 32), those who underwent other primary or rescue diagnostic PTCS (*n* = 16) or DOC (*n* = 2), those that were transferred from other hospitals (*n* = 2), and those with a short follow-up period of less than 6 months (*n* = 13) were all excluded. In total, 276 patients were included in the analyses and divided into a novel forceps group (*n* = 130) and a conventional forceps group (*n* = 146).

Table 1 presents the patients’ baseline characteristics according to each group. The novel forceps group had a mean age of 69.7 years, and 52.3% of patients were men. The conventional forceps group had a mean age of 71.8 years, and 59.6% of patients were men. The novel forceps group had a significantly higher proportion of extrahepatic strictures (80.8% vs. 69.2%, *p* < 0.001), although there was no significant inter-group difference in stricture length or EHD diameter. Among the proportion of methods of opening the major papilla, current endoscopic sphincterotomy on the same endoscopic session as performing endobiliary biopsy was the most frequent, without significant difference (novel forceps vs. conventional forceps; 80.8% vs. 69.9%, *p* = 0.154). There were no significant inter-group differences in terms of procedure-related AEs, such as bleeding, pancreatitis, or cholangitis. The novel forceps provided a histologic sample of optimal quality for pathologic examination in 129 cases (99.2%), compared to 137 cases (93.8%) using the conventional forceps, with a significant difference (*p* = 0.038). The most common final diagnosis in both groups was bile duct cancer (novel forceps: 46.2%, conventional forceps: 67.8%). The number of histological specimens was not significantly different between the novel and conventional forceps groups (3.3 ± 1.4 vs. 3.5 ± 1.6, *p* = 0.343).

### 3.2. Diagnostic Performance of Each Forceps for Endobiliary Biopsy

The diagnostic performance was calculated in two different ways according to the definition of malignancy for endobiliary biopsy. When the initial diagnosis by endobiliary biopsy only included positive malignancy, the novel forceps had 66.4% sensitivity, 100.0% specificity, a PPV of 100.0%, and an NPV of 42.6%, while the conventional forceps had 49.2% sensitivity, 100.0% specificity, a PPV of 100.0%, and an NPV of 29.9% (Table 2). The diagnostic accuracies were 73.1% for the novel forceps and 58.2% for the conventional forceps. When the initial diagnosis by endobiliary biopsy included suspected and positive malignancy, the novel forceps provided improved diagnostic performance (81.7% sensitivity, 100.0% specificity, a PPV of 100.0%, and an NPV of 57.8%) with an accuracy of 85.4%, while the conventional forceps provided a marginally improved diagnostic performance (61.7% sensitivity, 100.0% specificity, a PPV of 100.0% and an NPV of 36.1%) with an accuracy of 68.5%.

### 3.3. Variables that Were Associated with Diagnostic Accuracy

Univariable and multivariable analyses were performed using logistic regression models to identify factors associated with diagnostic accuracy (Table 3). Only novel forceps use was significantly associated with an accurate diagnosis in the univariable analysis (odds ratio (OR): 2.70; 95% confidence interval (CI): 1.49–5.00) and the multivariable analysis (OR: 2.70; 95% CI: 1.52–5.00).

The results of the univariable and multivariable analyses among only patients with malignant strictures are shown in Table 4. Novel forceps use was also significantly associated with an accurate diagnosis among these patients in the univariable analysis (OR: 2.78; 95% CI: 1.52–5.26; *P* = 0.001) and the multivariable analysis (OR: 2.94; 95% CI: 1.59–5.56; *P* < 0.001).

## 4. Discussion

The cytopathological diagnosis of biliary strictures can guide the decision to perform aggressive treatment for confirmed malignancy or avoid unnecessary surgery for benign disease [3]. The current options for ERCP-guided tissue acquisition in cases of biliary stricture are based on brush cytology or endobiliary forceps biopsy. A recent meta-analysis of nine studies revealed that the pooled sensitivity of endobiliary forceps biopsy was only 48% for diagnosing malignant biliary strictures (99% specificity) [13]. We evaluated novel slim biopsy forceps (Radial Jaw 4P, Boston Scientific) that were released in 2011 with various improvements, including a smaller shaft diameter (1.8 mm), improved flexibility, and a jaw angle that can reach 150° [14]. Furthermore, the jaws’ swing motion can also permit the acquisition of adequate tissue for a histological evaluation, even when faced with an oblique intraductal angle. Our results indicate that the novel slim biopsy forceps provided an adequate specimen for histological evaluation in 129 of 130 cases, while the same figure was 137 of 146 cases in the conventional forceps group. Furthermore, we observed a sensitivity value of 81.7% when the initial diagnosis by endobiliary biopsy included suspected and positive malignancy, which is higher than that in previous studies [2,3,19,22,23]. Thus, we conclude that novel forceps are a reasonable option for diagnosing biliary strictures.

The novel forceps provided better diagnostic accuracy than the conventional forceps, although the overall sensitivity of endobiliary biopsy remains insufficient. Thus, it is essential to identify factors associated with diagnostic accuracy, which can help guide the selection of the most appropriate sampling technique. Our results revealed that only novel forceps use was independently associated with an accurate diagnosis based on the multivariable logistic regression analysis. In contrast, Parsi et al. [24] reported that a positive diagnosis of biliary stricture was associated with older age and hyperbilirubinemia, and the mass was identified using a cross-sectional image. In addition, Naitoh et al. have reported that a positive diagnosis of malignancy after forceps biopsy was independently associated with bile duct cancer, a stricture length of ≥ 3 cm, and serum total bilirubin concentrations of ≥ 4 mg/dL, with longer strictures, likely providing greater sensitivity because they provide a greater area for contact with the forceps [7]. Moreover, higher serum bilirubin concentrations may be linked to greater biliary obstruction, which would permit a more perpendicular approach by the opened forceps. Nevertheless, we found that diagnostic accuracy was not significantly associated with age, stricture length, serum total bilirubin concentration, or bile duct cancer. It is also important to note that the etiology of malignant strictures can influence sensitivity, as pancreas, liver, gallbladder, or lymph node malignancies can theoretically compress the bile duct without direct invasion, which can lead to a secondary fibrous cicatricial stricture via the stromal reaction [25]. Furthermore, there are fewer tumor cells on the bile duct surface in cases of extrinsic neoplasms, relative to in cases of bile duct cancer [26]. Another prospective study clearly demonstrated that significantly better sensitivity was observed in cases with malignancies directly invading the bile duct, relative to cases with compression but not invasion (86% vs. 36%) [9]. Interestingly, our results indicate that the novel forceps provided greater sensitivity and diagnostic accuracy, despite having a significantly lower proportion of bile duct cancer (vs. the conventional forceps group). There is no clear explanation for the discrepancies between our findings and those of previous reports; however, they can be attributed to the following mechanisms. First, the novel forceps have jaws that can open to a relatively wide angle, which might permit better specimen acquisition. Second, the swing function might permit tangential biopsy even for fibrous cicatricial strictures, which could minimize the “bounce off” effect. Third, the thinner smooth shaft might permit biopsies even in cases with acute angulation of the duct [14].

Another noteworthy finding from our study was that AEs related to the endobiliary biopsy were rare. For example, immediate bleeding was not observed among the 130 patients in the novel forceps group, and was only observed for 2 out of 146 patients in the conventional forceps group. Schoefl et al. [12] also reported a very low rate of prolonged bleeding after forceps biopsy (one patient, 0.8%), and that case involved a patient with hilar cholangiocarcinoma who required a four-unit transfusion and the placement of a naso-biliary catheter. Pugliese et al. [11] reported one case of perforation in the common hepatic duct in a patient who underwent forceps biopsy at the distal edge of a stricture. This event might have been related to larger forceps jaw size, device stiffness, and/or a repeated biopsy of the same target stricture.

Our study has several limitations that might have influenced our final conclusions. First, the retrospective design is prone to bias related to incomplete data and patient selection. Second, the pathological results were determined by a single pathologist at each center, which may have introduced observer bias. Third, our methodology for confirming malignancy or benign disease has not been validated. Nevertheless, given the morbidity associated with the surgery and the fact that most patients presented with inoperable disease, long-term clinical follow-up and pathological evaluation are likely the most reasonable methods for assessing the diagnostic performance of endobiliary biopsy. A pathological report of “negative for malignancy” is generally thought to require a surgically resected specimen, although this may not be possible for all patients, especially patients with contraindications for surgery. Thus, our practice generally involves a clinical follow-up of ≥6 months with repeated imaging evaluations, and this approach is well accepted, though admittedly, not ideal.

In conclusion, endobiliary biopsy using novel forceps offered better diagnostic performance and more acceptable procedure-related adverse events relative to conventional forceps. We suggest that endoscopists should be aware that the novel forceps can facilitate easier endobiliary biopsy, even in cases with extrinsic compressive lesions, and offer better diagnostic performance. However, further prospective studies are needed to identify the optimal forceps size and characteristics that influence diagnostic accuracies, such as target lesion location and features.

## Figures and Tables

**Table 1:** Baseline patient characteristics of all patients who underwent ERCP with endobiliary biopsies.

	Novel Forceps (*N* = 130)	Conventional Forceps (*N* = 146)	*p*-Value
Age (year), (mean ± SD)	69.7 ± 12.5	71.8 ± 10.8	0.133
Sex (Male), *n* (%)	68 (52.3%)	87 (59.6%)	0.273
Level of structure, *n* (%)			0.038
− Extrahepatic duct	105 (80.8%)	101 (69.2%)	
− Perihilar	25 (19.2%)	45 (30.8%)	
Length of stricture (mm), (mean ± SD)	20.3 ± 7.5	18.9 ± 7.4	0.137
Diameter of EHD (mm), (mean ± SD)	12.4 ± 4.9	12.2 ± 5.5	0.688
Initial serum bilirubin (mg/dL), (mean ± SD)	5.9 ± 7.6	6.0 ± 6.7	0.949
Opening method of the major papilla, *n* (%)			0.154
− Current EST on the session of biopsy	105 (80.8%)	102 (69.9%)	
− Prior EST before the session of biopsy	20 (15.4%)	39 (26.7%)	
− Past EST	2 (1.5%)	2 (1.4%)	
− EPBD	3 (2.3%)	3 (2.1%)	
Adverse events, *n* (%)			
− Immediate bleeding	0 (0.0%)	2 (1.4%)	0.530
− Post procedural pancreatitis	6 (4.7%)	10 (6.8%)	0.604
− Post procedural cholangitis	3 (2.3%)	1 (0.7%)	0.529
− Bile duct perforation	0 (0.0%)	0 (0.0%)	>0.999
Number of biopsy (piece), (mean ± SD)	3.3 ± 1.4	3.5 ± 1.6	0.343
Procurement of adequate histologic sample, n (%)	129 (99.2%)	137 (93.8%)	0.038
Final diagnosis, *n* (%)			<0.001
− Benign stricture	26 (20.0%)	26 (17.8%)	
− Bile duct cancer	60 (46.2%)	99 (67.8%)	
− GB cancer	7 (5.4%)	0 (0.0%)	
− Metastatic cancer	4 (3.1%)	0 (0.0%)	
− Pancreatic cancer	33 (25.4%)	21 (14.4%)	

SD, standard deviation; EHD, extrahepatic duct; GB, gallbladder; EST, endoscopic sphincterotomy; EPBD, endoscopic papillary balloon dilatation.

**Table 2:** Diagnostic performance of each of the forceps for endobiliary biopsies by diagnosis 1 * and 2 ^#^.

	Novel Forceps		Conventional Forceps		Total Cohort	
	By Diagnosis 1 * N (%) (95% CI)	By Diagnosis 2 ^#^ N (%) (95% CI)	By Diagnosis 1 * N (%) (95% CI)	By Diagnosis 2 ^#^ N (%) (95% CI)	By Diagnosis 1 * N (%) (95% CI)	By Diagnosis 2 ^#^ N (%) (95% CI)
Sensitivity	69/104 (66.4%) (56.4–75.3)	85/104 (81.7%) (73.0–88.6)	59/120 (49.2%) (39.9–58.5)	74/120 (61.7%) (52.4–70.4)	128/224 (57.1%) (50.4–63.7)	159/224 (71.0%) (64.6–76.8)
Specificity	26/26 (100.0%) (86.8–100.0)	26/26 (100.0%) (86.8–100.0)	26/26 (100.0%) (86.8–100.0)	26/26 (100.0%) (86.8–100.0)	52/52 (100.0%) (93.2–100.0)	52/52 (100.0%) (93.2–100.0)
Accuracy	95/130 (73.1%) (64.6–80.5)	111/130 (85.4%) (78.1–91.0)	85/146 (58.2%) (49.8–66.3)	100/146 (68.5%) (60.3–75.9)	180/276 (65.2%) (59.3–70.8)	211/276 (76.5%) (70.1–81.3)
Negative predictive value	26/61 (42.6%) (36.2–49.3)	26/45 (57.8%) (47.7–67.3)	26/87 (29.9%) (26.3–33.7)	26/72 (36.1%) (31.1–41.5)	52/148 (35.1%) (31.8–38.7)	52/117 (44.4%) (39.5–49.5)
Positive predictive value	69/69 (100.00%)	85/85 (100.00%)	59/59 (100.00%)	74/74 (100.00%)	128/128 (100.00%)	159/159 (100%)

CI, confidence interval; * Diagnosis 1 was defined as only positive for malignancy from the initial endobiliary biopsy. # Diagnosis 2 was defined as positive and suspected for malignancy from the initial endobiliary biopsy.

**Table 3:** Variables for diagnostic accuracy according to univariable and multivariable logistic regression models.

Variables	Univariable Model		Multivariable Model	
	*p* Value	OR (95% CI)	*p* Value	OR (95% CI)
Novel forceps (vs. conventional forceps)	0.001	2.70 (1.49–5.00)	0.001	2.70 (1.52–5.00)
Age > 70 years	0.732	0.91 (0.51–1.58)		
Sex: Female	0.199	0.69 (0.40–1.21)	0.104	0.62 (0.35–1.10)
Level of stricture: Perihilar (vs. EHD)	0.413	0.77 (0.42–1.46)		
Length of stricture >3cm	0.967	1.03 (0.30–4.69)		
Total bilirubin > 4 mg/dL	0.075	0.60 (0.34–1.05)	0.097	0.61 (0.34–1.09)
EST * (vs. EPBD)	0.987	0.06 (0.01–2.00)		

OR, odds ratio; CI, confidence interval; EHD, extrahepatic duct; EST, endoscopic sphincterotomy; EPBD, endoscopic papillary balloon dilatation. * EST included current EST on the same session as performing endobiliary biopsy and prior EST at the previous endoscopic session within the same hospital stay, as well as post-sphincterotomy state, which it was assumed might be carried out at the unknown previous endoscopic session.

**Table 4:** Variables for diagnostic accuracy according to univariable and multivariable logistic regression models in malignant stricture.

Variables	Univariable Model		Multivariable Model	
	*p* Value	OR (95% CI)	*p* Value	OR (95% CI)
Novel forceps (vs. conventional forceps)	0.001	2.78 (1.52–5.26)	<0.001	2.94 (1.59–5.56)
Age > 70 years	0.965	0.99 (0.55–1.76)		
Sex: Female	0.127	0.64 (0.35–1.14)	0.076	0.58 (0.32–1.06)
Level of stricture: Perihilar (vs. EHD)	0.466	0.79 (0.42–1.52)		
Length of stricture > 3cm	0.753	1.24 (0.36–5.73)		
Diameter of EHD > 12mm	0.966	0.99 (0.55–1.78)		
Total bilirubin > 4 mg/dL	0.321	0.75 (0.42–1.33)		
EST* (vs. EPBD)	0.987	0.06 (0.01–2.00)		
Bile duct cancer (vs. non-bile duct cancer)	0.712	0.89 (0.48–1.69)		

OR, odds ratio; CI, confidence interval; EHD, extrahepatic duct; EST, endoscopic sphincterotomy; EPBD, endoscopic papillary balloon dilatation. * EST included current EST on the same session as performing endobiliary biopsy and prior EST at the previous endoscopic session within the same hospital stay, as well as post-sphincterotomy state, which it was assumed might be carried out at the unknown previous endoscopic session.
